# Ultrastructural Features, Immune Response, and Junctional Proteins in the Seminiferous Epithelium of SARS-CoV-2-Infected Mice

**DOI:** 10.3390/ijms27020691

**Published:** 2026-01-09

**Authors:** Salmo Azambuja de Oliveira, André Acácio Souza da Silva, Barry T. Hinton, Paulo Sérgio Cerri, Estela Sasso-Cerri

**Affiliations:** 1 Department of Morphology and Genetics, Federal University of São Paulo, São Paulo 04023-062, SP, Brazil; salmoazambujadeoliveira@gmail.com (S.A.d.O.); andre.acacio@unesp.br (A.A.S.d.S.); 2 Department of Cell Biology, School of Medicine, Virginia University, Charlottesville, VA 22903, USA; bth7c@virginia.edu; 3 Department of Morphology, Genetics, Orthodontics and Pediatric Dentistry, School of Dentistry, São Paulo State University (UNESP), Araraquara 14801-903, SP, Brazil; paulo.cerri@unesp.br

**Keywords:** COVID-19, Sertoli cell, cell death, nucleocapsid, immunofluorescence, electron microscopy

## Abstract

During the COVID-19 pandemic, the prevalence of death in men was higher than in women. Using transgenic mice expressing the human angiotensin-converting enzyme 2 (hACE2), we demonstrated that SARS-CoV-2 infects Leydig cells and uses its steroidogenic machinery for replication. This study investigates the impact of SARS-CoV-2 in the seminiferous epithelium of K18-hACE2 mice, focusing on the immune response, junctional proteins, and spermatogenesis. The seminiferous tubules (STs) and epithelial (EA) areas were measured. The number of Sertoli cells (SCs), spermatocytes, and damaged ST was quantified. Ultrastructural analysis was performed under transmission electron microscopy. Angiotensin II levels and immunolocalization of hACE2, spike, and nucleocapsid were evaluated. TUNEL and immunoreactions for Ki-67, TNF-α, INF-γ, iNOS, NF-κB, and Conexin-43 were performed and correlated with *Jam-α*, *Stat1*, *Stat3*, and *iNOS* expressions. hACE2, spike, and nucleocapsid immunolabeling were detected in the epithelium along with high angiotensin II levels in the infected mice. The infection caused a significant reduction in ST, EA, spermatocytes, SCs, Ki-67+ cells, Cx43 immunoexpression, and *Jam-a* expression. In the epithelium, TNF-α, IFN-γ, iNOS, and nuclear NF-κB immunolabeling increased along with *Stat1* upregulation. These findings, combined with the increased epithelial hACE2 and high angiotensin II levels, confirm epithelial responsiveness to the infection and explain the spermatogenic failure and impaired junctional proteins. The presence of viral particles, increased TNF-α immunolabeling, and apoptotic features in Sertoli cells suggests that these sustentacular cells are targets for viral infection in the epithelium, and, due to their extensive projections and ability to phagocytize dying infected germ cells, they may disseminate the viruses throughout the epithelium.

## 1. Introduction

SARS-CoV-2 is an enveloped virus [1] that causes COVID-19, a disease characterized by respiratory distress and clinical manifestations ranging from mild to severe [1]. Studies demonstrated that COVID-19 was more prevalent in males than in females, and men showed a high risk of developing severe COVID-19 [2,3]. According to the Genotype-Tissue Expression Project [4], the testis exhibits high expression of ACE2, a receptor for viral entry, detected in several testicular cells, including peritubular myoid cells, Sertoli cells (SCs), and germ cells [5,6,7]. Therefore, testes are susceptible to SARS-CoV-2, which causes testicular dysfunction [8,9,10], including reduced steroidogenesis [10] and spermatogenesis [7,11], leading to male infertility [8,9]. Additionally, in SARS-CoV-2-infected transgenic mice, the number of testicular macrophages increases, and Leydig cells polarize, shifting from a steroidogenic to an inflammatory profile in response to the infection. However, except for a few human ex vivo and postmortem studies [8,12,13] and in vivo and in vitro studies from transgenic animal models [13,14], the immune response of the seminiferous epithelium to SARS-CoV-2 infection, and the ultrastructural features of the infected Sertoli and germ cells have not been fully addressed.

SCs are susceptible to different viruses, such as Zika virus (ZIKV) [15,16], Marburg virus [17], mumps virus [18], and SARS-CoV-2 [8,9,19]. This last virus impairs SCs, disrupting the blood-testis barrier (BTB), and impairs spermatogenesis [18,20,21].

SCs are columnar cells that extend from the basement membrane to the lumen and emit cytoplasmic processes that surround and adhere to germ cells by cellular junctions [22]. The BTB, located between SC membranes at the basal compartment, is composed of the following junctional proteins: tight junction proteins (occludin, claudin, JAM-α, zonula occludens 1 (ZO-1)), gap junctions (connexin 43 and pannexins), and adhesion junctions (proteins, cadherins, β-catenin complex, desmosomes) [23,24]. Thus, the BTB provides an adequate microenvironment for developing germ cells while protecting germ cells from immune response and toxins [25,26]. Besides hormones, the BTB is also regulated by cytokines under physiological conditions; tumor necrosis factor-alpha (TNF-α), for example, produced by pachytene spermatocytes and round spermatids [27,28], binds to TNFR1 and TNFR2 in SCs and activates signaling pathways that regulate the passage of preleptotene/leptotene spermatocytes across the barrier as well as the release of spermatids during spermiation [29]. However, elevated levels of TNF-α impair the BTB proteins, such as Cx43 [16], claudin-11 [30], and Jam-α [31], leading to spermatogenic failure.

Interferons (IFNs) are the primary defense against viral infections [32,33], and exert anti-proliferative, immunomodulatory, and pro-apoptotic functions [34]. The binding of IFN-γ to its receptors IFN-γR1 and IFN-γR2 activates the Janus kinase/signal transducer and activator of transcription (JAK/STAT) pathway [34]. STAT1 is translocated to the nucleus and triggers the transcription of interferon-stimulated genes (ISGs), such as Interferon Regulatory Factor-1 (IRF1) [35,36], which play a key role in the host’s defense against viral infections [36,37]. An in vitro study has shown the expression of functional IFN-γR1 and IFN-γR2 as well as the expression of IRF-1 in SCs [38]. Moreover, studies have demonstrated that SCs are able to exert an antiviral response following infection [25,39,40].

The TNF-α and IFN-γ-mediated STAT1/IRF1 pathway induces inflammatory cell death through the production of inducible Nitric Oxide Synthase (iNOS) and Nitric Oxide (NO) [41]. iNOS transcription is regulated by different transcription factors, including Nuclear Factor-κB (NF-κB), IRF-1, and the STAT1 phosphorylated dimers [42]. In SARS-CoV-2-infected cells, IFN-γ induces iNOS-dependent production of NO, which promotes calcium efflux, resulting in reduced intracellular calcium levels, which inhibits furin, a calcium-dependent protease essential for SARS-CoV-2 replication in the respiratory tract [43,44]. A study using murine macrophages has shown that the synergism of TNFα and IFNγ induces iNOS and NO production through the activation of the JAK/STAT1/IRF1 signaling pathway [42]. In alveolar epithelial cells, IFNγ, TNFα, and IL-1β (produced by spike S1-activated macrophages) induce a significant *iNOS* expression through the JAK/STAT1/IRF1 pathway [45].

The K18-hACE2 mouse line intranasally infected with SARS-CoV-2 has been a robust model that develops the COVID-19-like disease [46,47]. Veras et al. (2023) [47] have demonstrated that male K18-hACE2 mice infected intranasally with this dosage have progressive weight loss and increased clinical scores, such as a marked increase in neutrophil extracellular traps in serum and lung, extensive lung injury with septal thickening and inflammatory infiltrates, and elevated pro-inflammatory cytokines and chemokines. They also demonstrated consistent heart and kidney lesions, and widespread apoptosis in lung tissue, pathological features compatible with severe COVID-19 in this model. In addition to these organs, testicular cells are also susceptible to SARS-CoV-2 infection [8,9,10] and immunologically responsive [10,48,49]. However, the ultrastructural and functional changes in the seminiferous epithelium following SARS-CoV-2 infection have been poorly explored. We used K18-hACE2 transgenic mice infected with an intermediate dose of SARS-CoV-2 to evaluate the impact of viral infection on Sertoli cells and germ cells, focusing on ultrastructural features, immune response, junctional proteins, and spermatogenic process.

## 2. Results

### 2.1. Immunolocalization of hACE2, Spike, and Nucleocapsid

**Figure 1:**
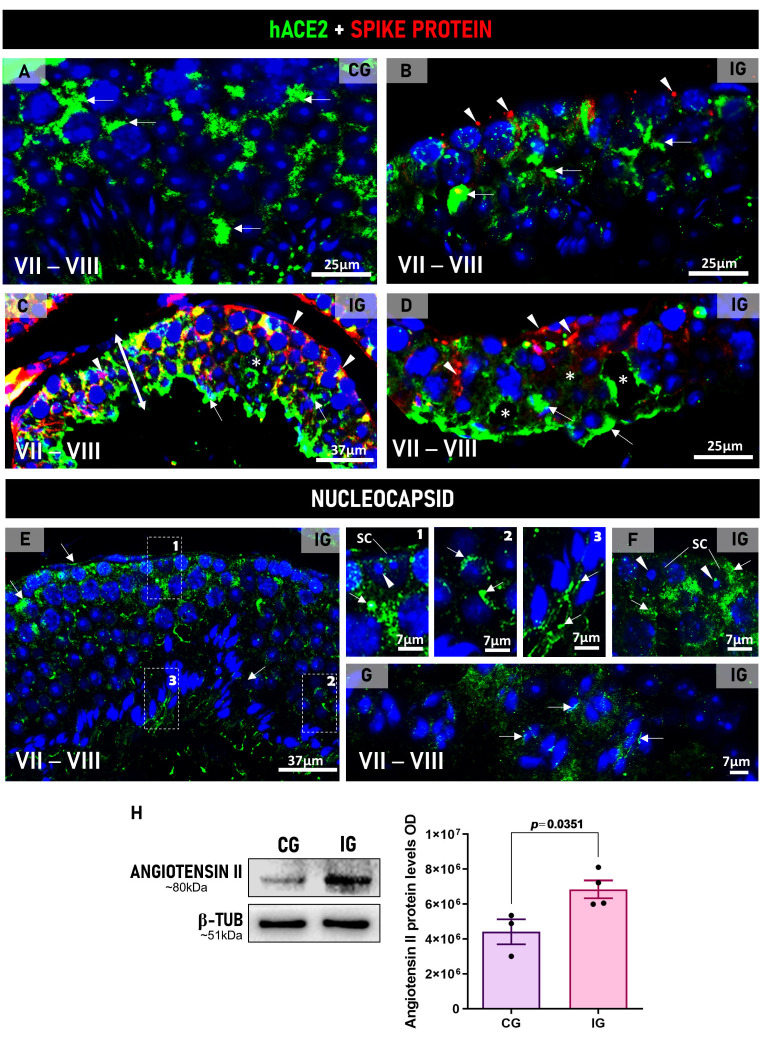
(**A**–**D**): Photomicrographs of testicular sections of animals showing double immunofluorescence for hACE2 and spike in animals from CG and IG (**A**–**D**). Nuclear staining with DAPI. In (**A**–**D**), sections of seminiferous tubules at stages VII–VIII show hACE2 immunoexpression (arrows) in both groups. In (**B**–**D**), in addition to hACE2 (arrows), spike immunolabeling (arrowheads) is observed throughout the seminiferous epithelium of IG. In (**C**,**D**), enhanced spike and hACE2 immunolabeling is observed in damaged regions of the seminiferous epithelium, which show reduced height (double headed arrow) and intraepithelial spaces due to loss of germ cells (*). (**E**–**G**)**:** Photomicrographs of testicular sections of animals showing immunofluorescence for nucleocapsid protein in animals from IG. Nuclear staining with DAPI. In (**E**), seminiferous tubules at stages VII–VIII show nucleocapsid immunolabeling (arrows) in Sertoli cells (inset 1), round spermatids (inset 2), and flagellum of elongate spermatids (inset 3). In (**F**,**G**), nucleocapsid immunoreaction is observed in Sertoli cells’ cytoplasm and elongate spermatids (arrows) of IG. (SC) Sertoli cell nucleus. SC nucleolus (arrowheads). (**H**): A weak angiotensin II signal is observed in CG when compared to a strong signal in IG. The β-tubulin signal is observed in both groups. A significant increase in angiotensin II optical density (OD) is observed in IG when compared to CG.

hACE2 immunolabeling was observed in the testicular cells of K18-hACE2 mice in CG and IG (Figure 1A–D). However, in IG, in addition to hACE2 immunolabeling (Figure 1B–D), spike immunoreaction was also observed throughout the seminiferous epithelium (Figure 1B–D), mainly in the tubules showing reduced epithelial height and intraepithelial spaces due to loss of germ cells (Figure 1C,D). Nucleocapsid immunolabeling was also detected in the seminiferous epithelium, including Sertoli cells (Figure 1E,F, spermatocytes, round (Figure 1E) and elongate (Figure 1E,G) spermatids, including the flagellum (Figure 1E).

The analysis by Western blot also showed a significant increase in angiotensin II protein levels (*p* = 0.0351) in IG in comparison to CG (Figure 1H).

### 2.2. Impact of SARS-CoV-2 Infection on the Seminiferous Tubules Histoarchitecture

**Figure 2:**
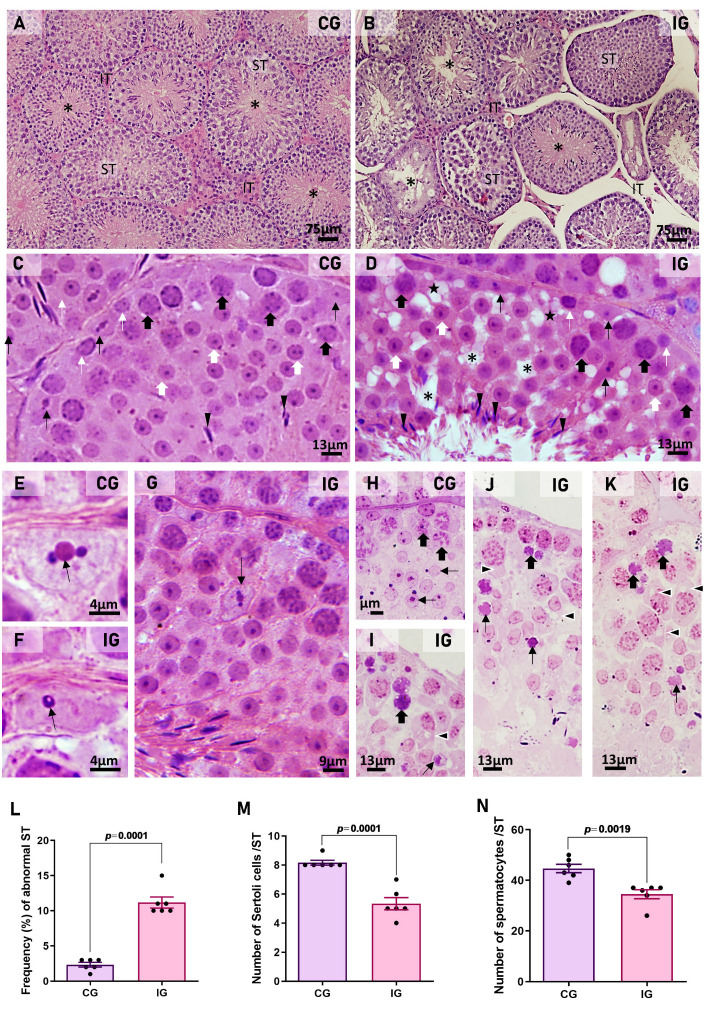
(**A**–**G**): Photomicrographs of testicular sections from CG and IG stained with H.E. In (**A**), normal seminiferous tubules (STs) show intact epithelium, whereas in (**B**), the tubules are smaller and show numerous intraepithelial spaces and a large lumen (*) compared to (**A**). Interstitial tissue (IT). In (**C**,**D**), regions of tubules showing spermatogonia (thin white arrows), Sertoli cell nuclei (thin black arrows), spermatocytes (thick black arrows), round spermatids (thick white arrows), and elongated spermatids (arrowheads). In (**D**), the epithelium exhibits intraepithelial spaces (*), a lack of spermatocytes (stars), and basophilic Sertoli cell nuclei (thin black arrows). In (**E**,**F**), Sertoli cell nuclei with a typical nucleolus (arrow). Note that in (**F**), the nucleus is irregular and more stained than in CG. In (**G**), a nucleus of a Sertoli cell is displaced from the basal epithelium (arrow). (**H**–**K**): Photomicrographs of semithin sections stained with toluidine blue**.** In (**H**), organized seminiferous epithelium shows normal germ cells: spermatocytes (thick arrow) and spermatids (thin arrow). In (**I**–**K**), atypical spermatogonia/spermatocytes (thick arrows) and spermatids (thin arrows) show cytoplasmic vacuoles (arrowheads) and strongly basophilic nuclei, indicative of cell death. (**L**): The number of abnormal STs is significantly higher in IG. (**M**): The number of Sertoli cells per ST is significantly lower in IG animals. (**N**): The number of spermatocytes per ST is significantly lower in IG animals.

In the testicular sections of animals from the CG, the seminiferous tubules (STs) showed regular outlines and organized seminiferous epithelium with typical concentric germ cell layers (Figure 2A,C) and typical SC nuclei exhibiting light basophilic staining and a prominent nucleolus (Figure 2C,E). However, in IG, the ST were apparently reduced and showed numerous intraepithelial spaces due to loss of germ cells (Figure 2B,D). Some SC nuclei exhibited irregular outlines and intense basophilia (Figure 2D,F,G) compared to CG (Figure 2C,E). SC nuclei in the adluminal compartment were also found (Figure 2G).

In CG, the semithin sections showed germ cells with typical nuclear morphology (Figure 2H), whereas in IG, intracytoplasmic vacuoles were usually found in the spermatocytes and round spermatids, and some of these cells showed an irregular and strongly basophilic nucleus, indicative of cell death (Figure 2I–K).

The frequency of abnormal ST increased significantly in IG (*p* = 0.0001) compared to CG (Figure 2L), and a significant decrease in the number of SC (*p* = 0.0001) and spermatocytes (*p* = 0.0019) was also observed in IG (Figure 2M,N).

### 2.3. Body and Testicular Weights, and Seminiferous Tubule Areas

The body weight of animals from IG was reduced compared to CG (*p* = 0.0300). However, absolute testes weight showed no significant difference (*p* = 0.1349) in the animals from IG in comparison with CG (Table 1). A significant reduction in total tubular (*p* = 0.0097) and epithelial (*p* = 0.0019) areas was detected in the testes of animals from IG. Due to the epithelial area reduction, the luminal area increased significantly (*p* = 0.0065) in the infected animals, as shown in Table 1.

### 2.4. Infection by SARS-CoV-2 Impairs Sertoli Cells and Causes Germ Cell Death

**Figure 3:**
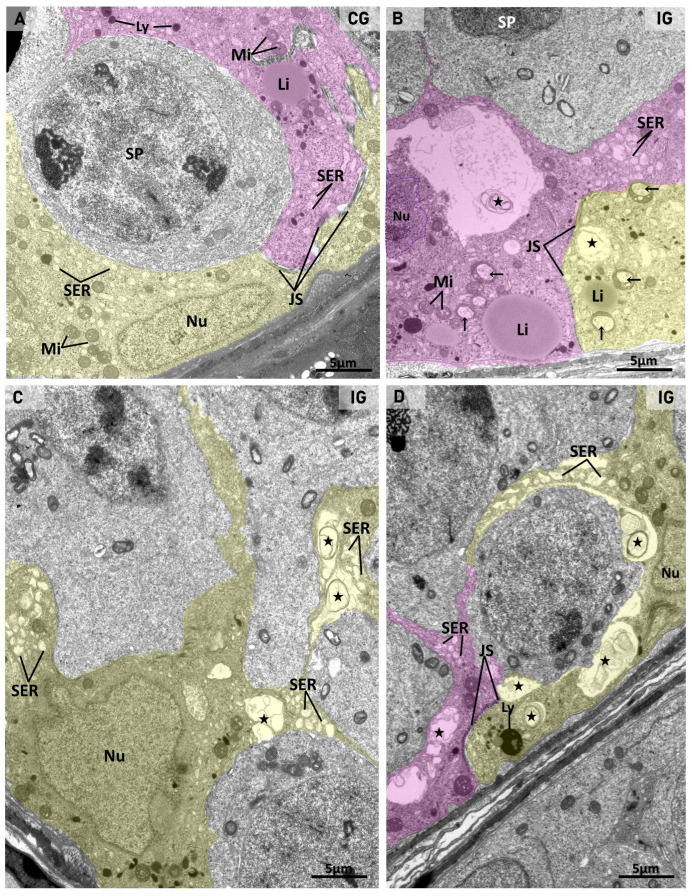
(**A**–**D**): Electron micrographs of the basal compartment of seminiferous tubules showing Sertoli cells in CG (**A**) and IG (**B**–**D**). In A, two Sertoli cells (yellow and pink colors), attached to each other by junctional specializations (JS), show a basal nucleus (Nu) and cytoplasm surrounding a spermatocyte (SP). Lipid droplets (Li), mitochondria (Mi), lysosomes (Ly), and smooth endoplasmic reticulum (SER) are observed in the cytoplasm. In (**B**–**D**), Sertoli cells of animals from IG show larger mitochondria (Mi) than in (**A**), and contain vacuoles with a thin granular material (arrows). Nucleus (Nu). Clusters of large smooth endoplasmic reticulum cisternae (SER) are observed; some of them are next to membranous vesicles (stars). In (**C**), note the irregularly outlined nucleus (Nu). In (**D**), the interface of the two juxtaposed Sertoli cells (yellow and pink colors) shows intact integrity of the junctional specializations (JS).

**Figure 4:**
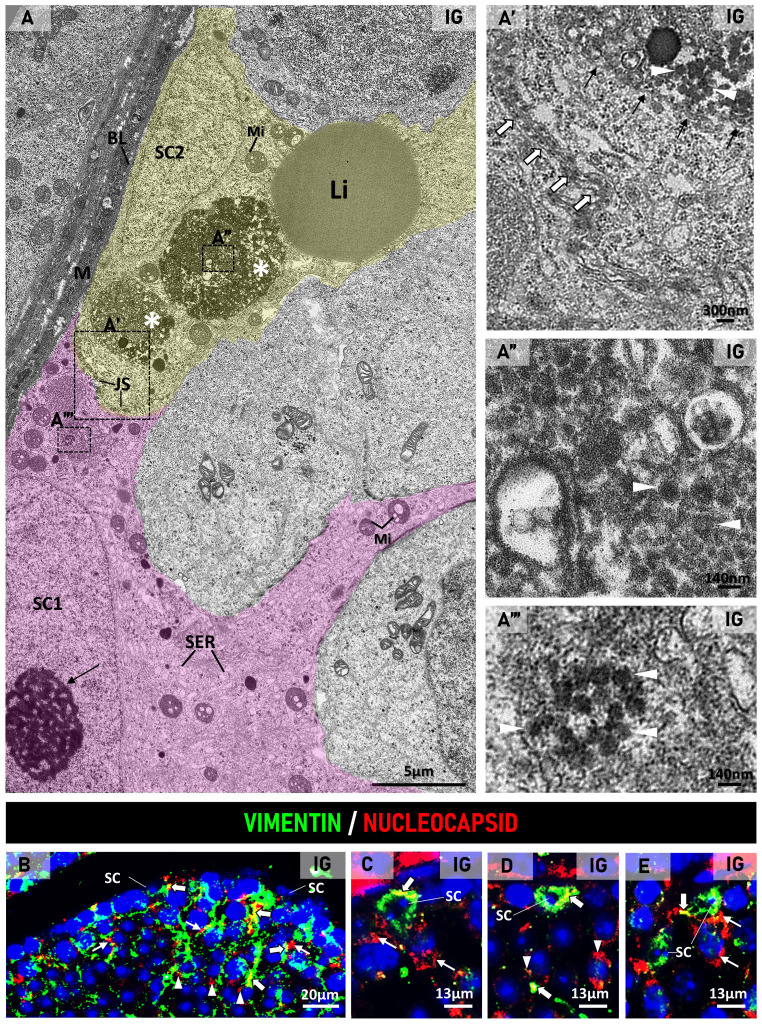
(**A**): Electron micrograph of the basal region of the seminiferous epithelium showing infected Sertoli cells. In (**A**), two juxtaposed Sertoli cells (SC1 and SC2) show a typical nucleolus (SC1; arrow), smooth endoplasmic reticulum (SER), mitochondria (Mi), and a lipid droplet (Li) in the cytoplasm. In the SC1/SC2 interface, junctional specializations of BTB (JS and (**A′**), high magnification; white arrows) are observed. In SC2, assembled viral particles are within large vesicles (asterisks) delimited by membrane ((**A′**) high magnification; black arrows). A clump of viral particles is also observed in SC1 cytoplasm (**A‴**). Under high magnification (**A′**,**A″**,**A‴**), note assembled viral particles measuring around 140 nm (white arrowheads). (BL) basal lamina. (M) myoid cell. (**B**–**E**): Photomicrographs of seminiferous tubules showing double immunofluorescence for vimentin (green) and nucleocapsid (red). Nuclear staining with DAPI. In (**B**–**E**), vimentin-immunolabeled cytoplasmic projections of Sertoli cells (green) are also positive for nucleocapsid (yellow; thick arrows), and some of them are next to nucleocapsid- immunolabeled spermatocytes (thin arrows) and spermatids (arrowheads). Sertoli cell nucleus (SC).

**Figure 5:**
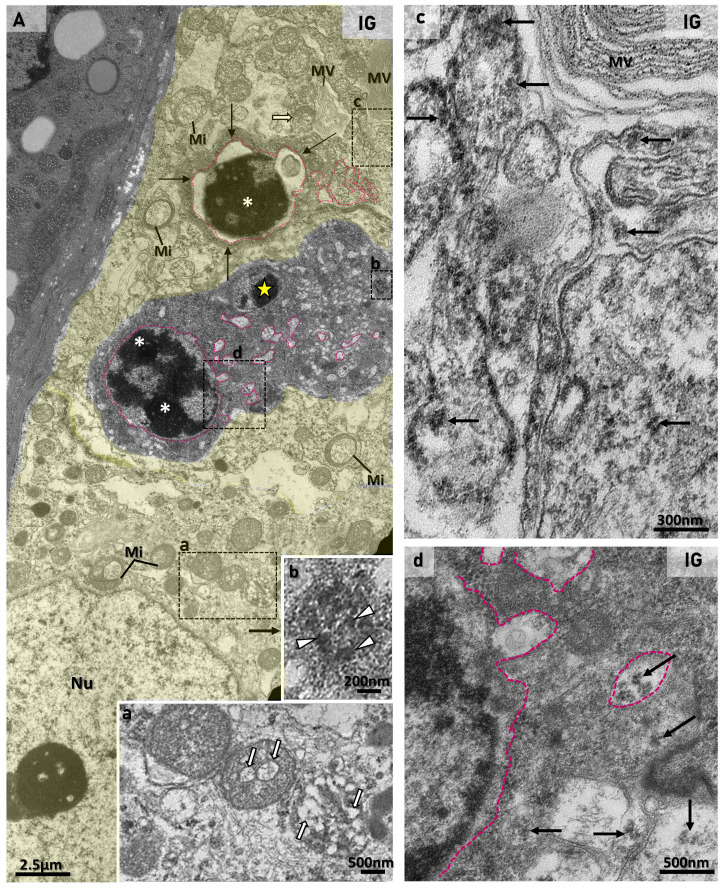
(**A**) Electron micrograph of the basal region of the seminiferous epithelium. An infected Sertoli cell (yellow color) surrounds dying germ cells whose nucleus shows condensed chromatin (asterisks). A detached nuclear portion with condensed chromatin is observed in the germ cell cytoplasm (star). In the SC cytoplasm, swollen mitochondria exhibiting only a large vacuole (Mi), containing a thin granular material, are observed. Some mitochondria show dilated cristae (inset (**a**); white arrows) whose fusion gives rise to swollen mitochondria with only a vacuole. Membranous whorls (MW) are observed in the SC cytoplasm. In the dying germ cells, note nuclear membrane dilations (pink line) protruding towards the cytoplasm (black arrows). Some protrusions extend throughout the cytoplasm, forming convoluted vesicles (delimited by the pink line). In the cytoplasm, a cluster of viral particles (nucleocapsid) is observed (inset-(**b**); arrowheads). In (**c**), high magnification of the Sertoli cell cytoplasm, note numerous nucleocapsid proteins (arrows). In (**d**), a high magnification of the interface between a dying germ cell and Sertoli cell shows nucleocapsid proteins (arrows) in both cells, including in the nuclear membrane-derived convoluted vesicles (pink line).

**Figure 6:**
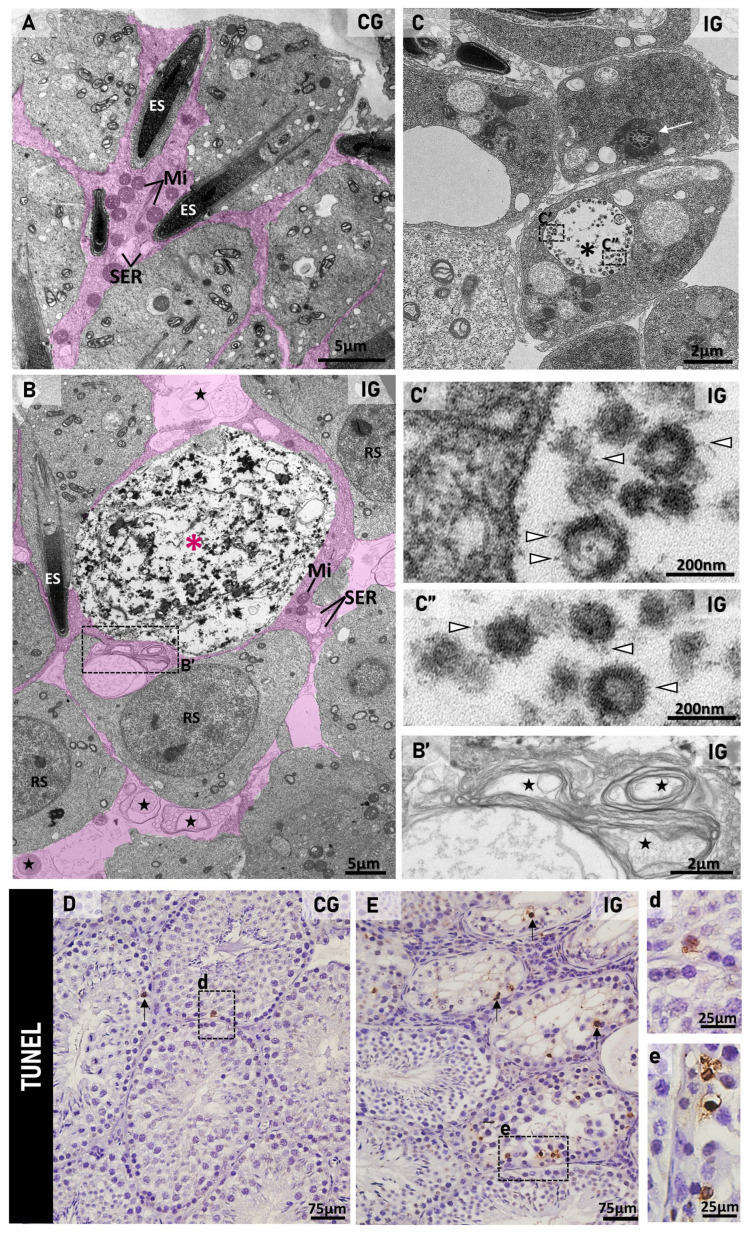
(**A**–**C**)**:** Electron micrographs of the adluminal compartment of seminiferous tubules in CG (**A**) and IG (**B**,**C**). In (**A**,**B**), Sertoli cell cytoplasmic projections (pink color), containing mitochondria (Mi) and smooth endoplasmic reticulum (SER), surround normal elongate (ES) and round (RS) spermatids as well as a dying germ cell ((**B**); pink asterisk). In (**B**), vacuolated portions of SC cytoplasmic projections show only membranous vesicles (stars). In (**B′**), high magnification of (**B**), note a multi-layered membranous vesicle (stars). In (**C**), the adluminal region of the epithelium shows cytoplasmic portions of elongating spermatids; note the flagellum (centriole) in cross section (white arrow). In another cell, a large vesicle (asterisk) contains numerous enveloped/assembled viral particles. In the black boxes and at high magnification (**C′**,**C″**), enveloped viruses measuring ~160 nm are observed, surrounded by spike proteins (arrowheads). (**D**,**E**): Photomicrographs of testicular sections of animals from CG and IG subjected to the TUNEL method. In (**E**) (IG), several TUNEL-positive germ cells (arrows) are observed in the seminiferous epithelium in comparison to (**D**) (CG). In (**d**,**e**) (high magnification of outlined areas), germ cells with TUNEL-positive nucleus (brown-yellow) are observed.

The analysis of the seminiferous epithelium under TEM showed SCs exhibiting a nucleus with a typical nucleolus, smooth endoplasmic reticulum, mitochondria, lysosomes, and lipid droplets either in CG or IG (Figure 3A–C, Figure 4A, Figure 5A and Figure 6A). In both groups, the interface of juxtaposed SC membranes showed junctional specializations forming the BTB (Figure 3A,B,D and Figure 4A). In IG, some SCs showed large mitochondria with vacuoles containing a thin granular material (Figure 3B and Figure 5A), and large endoplasmic reticulum cisternae, which were also found next to membranous vesicles (Figure 3B–D). In some SCs, cytoplasm, assembled viral particles, measuring around 140 nm, were also found within vesicles delimited by membrane (Figure 4A and Figure 5A).

The double immunofluorescence reaction for the detection of vimentin (Sertoli cell marker) and nucleocapsid confirmed the presence of nucleocapsid in the Sertoli cell cytoplasmic projections, which were also next to infected germ cells (Figure 4B–E).In the damaged SCs, the cytoplasmic projections were vacuolated and showed membranous or multi-layered membranous vesicles as well as membranous whorls (Figure 3B–D, Figure 5A and Figure 6B). Damaged and/or infected SC projections surrounded either normal or dying germ cells (Figure 3B–D, Figure 4A, Figure 5A and Figure 6B).

Whereas the normal ultrastructure of germ cells was observed in CG (Figure 3A and Figure 6A), in IG, germ cells showing typical features of cell death were usually found. These cells showed electron-opaque condensed chromatin as well as nuclear fragments in the cytoplasm, indicative of apoptosis (Figure 5A and Figure 6B). These dying germ cells showed dilations in the nuclear membrane and viral particles (nucleocapsid proteins) within cytoplasmic vesicles (Figure 5A and Figure 6B). In the adluminal regions of the epithelium, elongating spermatids also showed large vesicles containing numerous assembled/enveloped viral particles with spike proteins, measuring 140 nm to 160 nm (Figure 6C).

In CG, scarce TUNEL-positive germ cells were found (Figure 6D) in contrast to the numerous TUNEL-positive germ cells observed in the seminiferous epithelium of IG (Figure 6E).

### 2.5. Viral Infection Increases Cytokines and Impairs Junctional Molecules and Spermatogenic Activity

**Figure 7:**
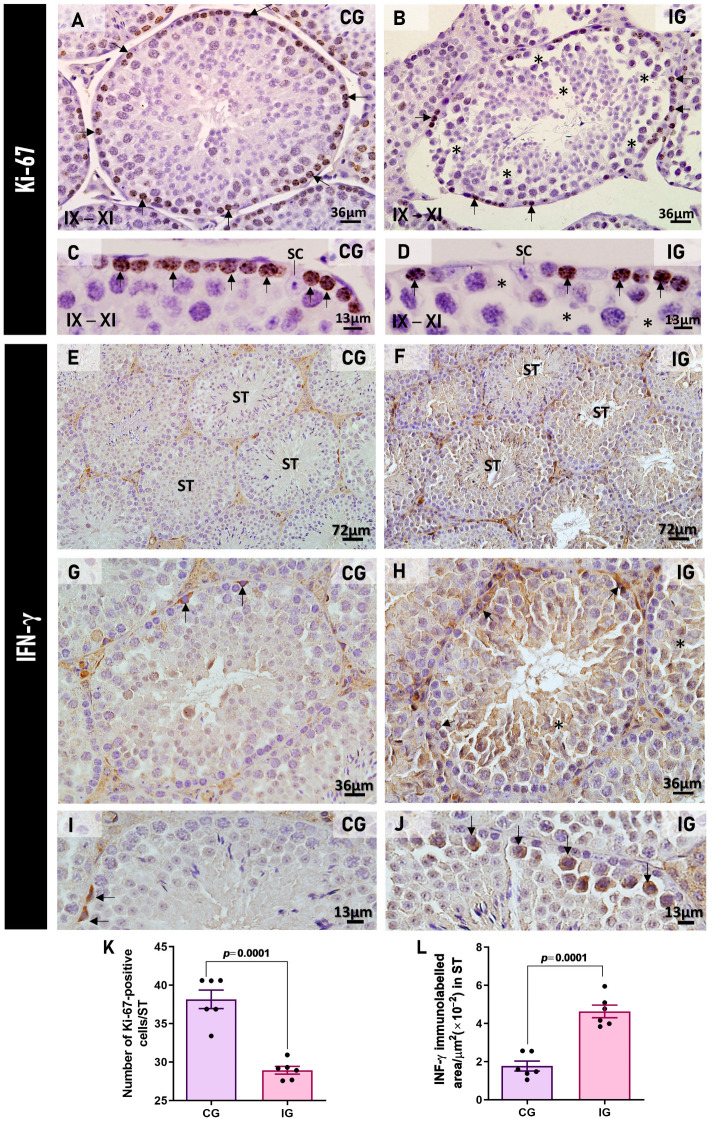
(**A**–**D**): Photomicrographs of testicular sections of animals from CG and IG submitted to Ki-67 immunohistochemistry. In (**A**,**C**), seminiferous tubules at stages IXXI show several Ki-67 immunopositive cells (black arrows) in CG. In (**B**,**D**), a few Ki-67-immunopositive cells (arrows) are observed in the epithelium of IG. Intraepithelial spaces (asterisks). In (**C**,**D**), Sertoli cell nuclei (SC). (**E**–**J**): Photomicrographs of testicular sections of animals from CG and IG submitted to IFN-γ immunohistochemistry. In (**E**,**G**,**I**), weak immunostaining is observed in the seminiferous tubules (ST). In (**G**,**I**), occasional immunolabeled spermatogonia (arrows) are observed. In (**F**,**H**,**J**), enhanced immunostaining is observed in the ST. In (**H**), in addition to the basal compartment (arrows), a diffuse immunolabeling is also observed through the epithelium of IG (asterisks). In (**J**), evident IFN-γ-positive spermatocytes (arrows) are observed. (**K**): The number of Ki-67-immunopositive cells is significantly higher in CG compared to IG. (**L**): The IFN-γ immunofluorescent area is significantly increased in IG compared to CG.

Reduction in Ki-67-immunopositive spermatogonia and spermatocytes was observed in IG (Figure 7B,D), in contrast to numerous immunolabeled cells in CG (Figure 7A,C). The quantitative analysis showed a significant decrease in the number of Ki-67-immunopositive cells/ST (*p* = 0.0001) in IG when compared to CG (Figure 7K).

In the testicular sections of animals from both CG and IG, IFN-γ immunolabeling was usually found in the spermatogonia (Figure 7E,G,I). However, in IG, strong immunolabeling was observed in the germ cells of the basal compartment (Figure 7F,H), mainly spermatocytes (Figure 7J). The IFN-γ immunofluorescent area increased significantly (*p* = 0.0001) in IG in comparison to CG (Figure 7L).

**Figure 8:**
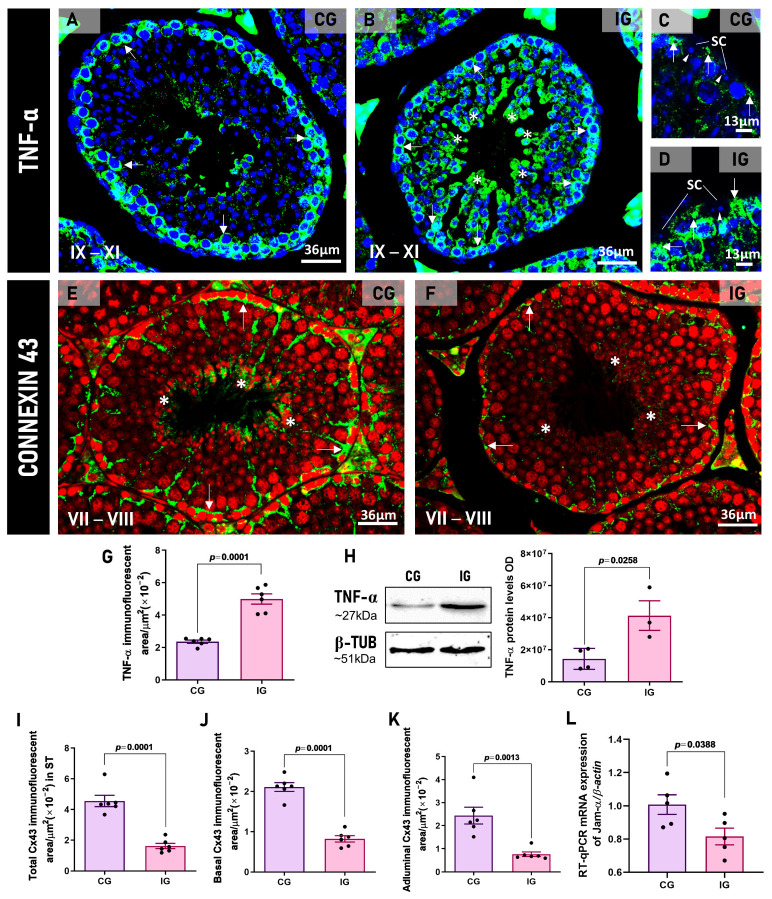
(**A**–**D**): Photomicrographs of testicular sections showing TNF-α immunofluorescence in CG (**A**) and IG (**B**–**D**). Nuclear staining with DAPI. In (**A**,**B**), seminiferous tubules at stages IX–XI show TNF-α immunofluorescence in the spermatocytes (arrows). In (**B**), strong TNF-α immunofluorescence is noted in spermatocytes (arrows) and spermatids (*). Under high magnification (**C**,**D**), TNF-α immunolabeling is observed in Sertoli cells (arrows). (SC) Sertoli cell nucleus; SC nucleolus (arrowheads). (**E**,**F**): Photomicrographs of testicular sections showing Connexin 43 (Cx43) immunofluorescence in CG and IG. Nuclear staining with propidium iodide. In (**E**,**F**), note the strong Cx43 immunolabeling in the basal (arrows) and adluminal (asterisks) compartments in CG when compared to IG. (**G**): The TNF-α immunofluorescent area is significantly increased in IG compared to CG. (**H**): A weak TNF-α signal is observed in CG when compared to a strong signal in IG. The β-tubulin signal is observed in both groups. A significant increase in TNF-α optical density (OD) is observed in IG compared to CG. (**I**–**K**): In IG, the Cx43 immunofluorescent area decreased significantly in the seminiferous epithelium (**I**), including in basal (**J**) and adluminal (**K**) compartments. (**L**): A significant decrease in the mRNA expression of *Jam-α* is observed in IG when compared to CG.

TNF-α immunoreaction was detected in the seminiferous epithelium of the testicular sections of animals from both CG and IG (Figure 8A–D). However, whereas in CG, the immunolocalization was specifically located in the basal region, mainly spermatocytes (Figure 8A), in IG, in addition to spermatocytes, an evident immunoreaction was also observed in SCs and spermatids (Figure 8B–D). The TNF-α immunofluorescent area increased significantly (*p* = 0.0001) in IG in comparison to CG (Figure 8G). The analysis by Western blot confirmed the significant increase in TNF-α protein levels (*p* = 0.0258) in IG in comparison to CG (Figure 8H).

Immunoexpression of Connexin 43 (Cx43) was observed in the seminiferous tubules (stages VII–VIII) in both groups (Figure 8E,F). However, an intense Cx43 immunofluorescence was observed throughout the basal and adluminal compartments of the seminiferous epithelium of CG (Figure 8E), whereas a weak and punctate immunoexpression was observed only in the basal compartment of the seminiferous epithelium of IG (Figure 8F). The immunofluorescent area of Cx43 was reduced significantly in the seminiferous epithelium of animals from the IG. This reduction was detected either in the basal or adluminal compartments in IG (*p* = 0.0001 and *p* = 0.0013) compared to CG (Figure 8I–K). The *Jam-α* gene expression also decreased significantly (*p* = 0.0388) in the animals from the IG (Figure 8L).

**Figure 9:**
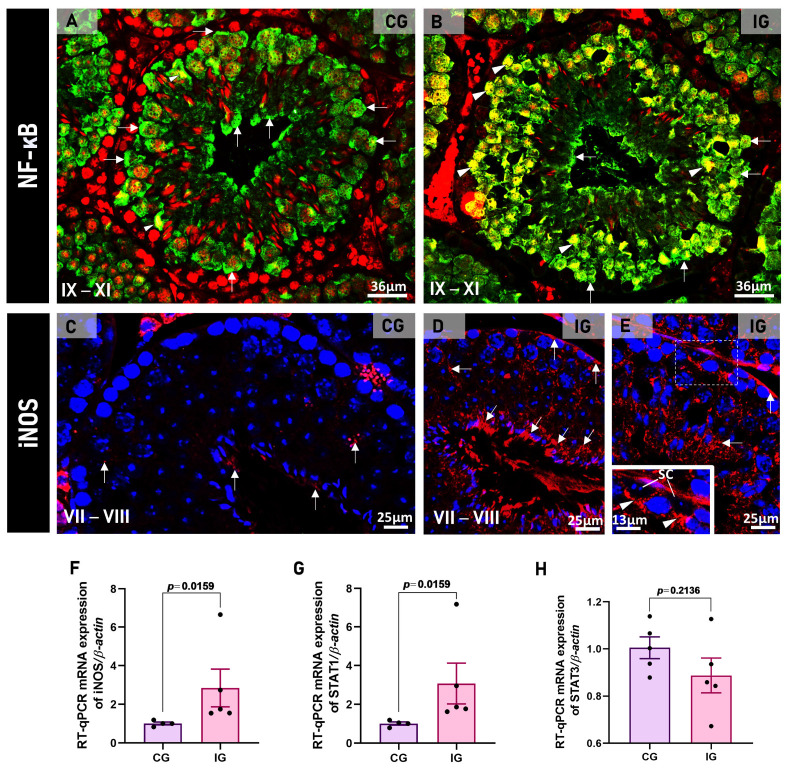
(**A**,**B**): Photomicrographs of testicular sections submitted to NF-kB immunofluorescence (green) in CG and IG. Nuclear staining with propidium iodide in red In (**A**,**B**), NF-kB immunoexpression is observed in the germ cells’ cytoplasm (green fluorescence; arrows); however, in IG (**B**), the germ cell nuclei are also stained (yellow immunofluorescence; arrowheads). (**C**,**E**): Photomicrographs of testicular sections showing iNOS immunofluorescence (red) in CG and IG. Nuclear staining with DAPI. In (**D**,**E**), note an intense iNOS immunoexpression (arrows) throughout the seminiferous epithelium in comparison to weak immunolabeling in the epithelium of CG (**C**). In (**E**), iNOS immunoreaction in Sertoli cells (inset). (SC) Sertoli cell nuclei. SC nucleolus (arrowheads). (**F**–**H**): The *iNOS*, *Stat1* mRNA expression increased significantly in the animals from the IG, whereas *Stat3* expression was similar to that of the CG.

Either in CG or IG, evident NF-kB immunoexpression was observed in the cytoplasm of germ cells (Figure 9A); however, in IG, a strong immunolabeling was also detected in the germ cell nuclei, which showed yellow fluorescence (Figure 9B). Moreover, a strong iNOS immunoexpression was also observed in the germ cells and Sertoli cells of the testes of animals from the IG (Figure 9D,E) in comparison to the CG (Figure 9C). Either iNOS or *Stat1* gene expression increased significantly (*p* = 0.0001 and *p* = 0.0159, respectively) in IG (Figure 9F,G), whereas no difference (*p* = 0.2136) was detected in the expression of *Stat3* between CG and IG (Figure 9H).

## 3. Discussion

The viral receptor hACE2 was detected in the seminiferous epithelium of both infected and non-infected transgenic K18-hACE2 mice. Moreover, in IG, the elevated angiotensin II levels associated with the presence of viral particles and/or assembled viruses in Sertoli cells (SCs) and germ cells corroborate the susceptibility of the epithelium to SARS-CoV-2 infection. The number of SCs and germ cells decreased in association with damage to BTB junctional specializations, low mitotic/meiotic activity, and germ cell death. These changes may be a consequence of the increased iNOS production mediated by the TNF-α and IFN-γ synergic effect in the seminiferous epithelium. The enhanced nuclear NF-kB immunoexpression in germ cells, along with the high STAT1 mRNA levels, corroborates these findings. In addition, the presence of SCs containing SARS-CoV-2 proteins, assembled viral particles, and enhanced TNF-α concentration in the cytoplasm confirms that this cell is a target for viral infection in the seminiferous epithelium, exerts an immune response, and, due to its intricate cytoplasmic projections in close contact with numerous germ cells, this sustentacular cell may also spread the viruses to germ cells located in the basal and adluminal compartments.

### 3.1. SARS-CoV-2 Infects the Seminiferous Epithelium and Increases Angiotensin II and hACE2 Expression

Our results confirmed the immunoexpression of hACE2 in the seminiferous epithelium of the transgenic K18-hACE2 mice, reinforcing the susceptibility of this epithelium to SARS-CoV-2 infection. It is important to emphasize that although the K18 promoter drives overexpression of hACE2 in the testes of transgenic mice, enhancing the viral tropism to the seminiferous tubules, the intrinsic ACE2 expression level in the seminiferous epithelium of the human testis is notably high and comparable to that achieved in the transgenic model [46]. Moreover, the consistency of our results with the existing literature [5,7,8,49,50] validates the clinical relevance of the present study.

Both spike and nucleocapsid proteins were detected in the seminiferous epithelium, and the ultrastructural analyses confirmed the presence of viral particles in the SCs and germ cells. Transmission electron microscopy is a unique method to identify assembled viruses in cells, confirming SARS-CoV-2 replication outside the respiratory tract [51]. In our current study, the identification of the assembled/enveloped viral particles under TEM was based on the presence of nucleocapsid proteins surrounded by an envelope, the size of the assembled virus (~150 nm), the presence of assembled virus within membrane vesicles [10,51,52], as well as the identification of isolated nucleocapsid proteins (spread or in clusters) in the cytoplasm [10]. Based on these features, we found viral particles, including assembled virus, in SCs, spermatocytes, and spermatids in IG animals. Similar findings were also observed in the seminiferous epithelium of *post-mortem* testes from COVID-19 patients [8,9,11]. In addition to the viral ultrastructural features, the presence of membranous vesicles is also indicative of SARS-CoV-2 replication and assembly [51]. It is known that, after entering the host cell, the genomic viral RNA induces the replication–transcription complex, which induces an extensive remodeling of intracellular membranes, forming the replication membranous web, where viral RNA is generated. The nucleocapsid protein associated with RNA invaginates into the ERGIC membrane, containing spike proteins, and gives rise to new viruses within vesicles [51]. In our current study, under TEM, the SCs showed dilated smooth ER cisternae, and some of them were next to large membranous vesicles, confirming remodeling of ER and, hence, the viral replication in these cells. Another marked feature of viral infection is the presence of swollen and vacuolated mitochondria with dilated cristae. The characteristic mitochondrial swelling and vacuolation observed in SARS-CoV-2-infected cells is hypothesized in the literature [52] to facilitate viral replication, as mitochondrial-derived vesicles (MDVs), shed during organelle stress, may function as precursors to the virus-induced double-membrane vesicles (DMVs). Thus, the presence of swollen and vacuolated mitochondria with dilated cristae in the SCs of IG is another robust morphological feature that confirms Sertoli cell infection by SARS-CoV-2. In addition to mitochondria, the nuclear membrane is also involved in the remodeling of the SARS-CoV-2-infected cells. In infected submandibular gland cells, nucleocapsid proteins and/or viral particles were found in the nuclear membrane-derived vesicles [53]. Similarly to these findings, the cytoplasm of dying germ cells showed nuclear membrane dilations, which were protruding towards the cytoplasm, forming convoluted vesicles containing nucleocapsid proteins. These findings reinforce the participation of the nucleus in the viral replication/formation, as previously demonstrated [53].

Studies have shown that ACE2 expression is upregulated by the activation of pathways triggered by viral sensors as well as by cytokines, such as TNF-α [54,55,56]. In fact, the viral sensor RIG-1 was overexpressed in the testes of the animals from the IG [10], and either intense TNF-α immunolabeling in the seminiferous epithelium or high testicular TNF-α protein levels were observed in the IG. These findings, in association with the presence of viral particles in the epithelium, corroborate the increased hACE2 observed in our study.

Some studies have shown elevated angiotensin II levels in COVID-19 patients compared to healthy controls [57,58,59]. Moreover, critically ill patients showed significantly higher angiotensin II levels than those with mild symptoms [58,59], pointing to angiotensin II as a potential biomarker for COVID-19 morbidity [57]. In addition, in vitro studies using SARS-CoV-2 pseudoviruses showed that increased levels of angiotensin II contribute to cell infection with SARS-CoV-2 [60,61]. In our study, the testes of the infected mice showed increased angiotensin II protein levels along with increased hACE2 expression, suggesting that the synergism between these proteins could have contributed to testicular SARS-CoV-2 infection. In a previous study, we demonstrated a direct correlation between hACE2 and spike immunolocalization in the epididymis of SARS-CoV-2-infected mice, indicating that the infection itself stimulates hACE2 expression, allowing more viruses to infect cells [56]. Our findings reinforce this idea since intense spike immunolabeling was colocalized with enhanced hACE2 immunostaining in the seminiferous tubules of IG. In endothelial cells, the synergy between TNF-α and IFN-γ enhances the expression of SARS-CoV-2 entry receptors, such as ACE2, and hyperactivates the JAK/STAT1 pathway [62]. It is known that STAT1 activation is typically confirmed by detecting its phosphorylated form, which translocates to the nucleus [63]. However, in the current study, we evaluated STAT1 mRNA levels as an attempt to corroborate the TNF-α and IFN-γ synergistic effect, as confirmed by increased iNOS and nuclear NF-kB immunolabeling. Therefore, the *Stat1* upregulation in association with the enhanced TNF-α and IFN-γ concentrations observed in the seminiferous tubules of IG may support the high hACE2 upregulation.

Studies have demonstrated double-membrane vesicles containing SARS-CoV-2 particles in SCs [8,9]; however, the ultrastructural changes caused by this viral infection in these cells have not yet been addressed. In the present study, the infected SCs showed large mitochondria, dilated endoplasmic reticulum cisternae, and multi-layered membranous vesicles, typical features of reticulum remodeling, induced during SARS-CoV-2 infection for viral replication [51]. Moreover, the nucleocapsid and spike proteins’ immunolocalization in the SCs corroborates the ultrastructural findings, including the presence of vacuolated cytoplasmic projections; these findings support the significant reduction in the number of these cells, confirming their susceptibility to SARS-CoV-2. Studies have demonstrated the viral tropism for SC as well as its immune response to viral infections [25,39,40]. The present study showed an intense immunoexpression of NF-kB and TNF-α in the SCs of IG, corroborating these previous findings and confirming a pro-inflammatory immune response of SCs to SARS-CoV-2 infection.

SC is a columnar cell laid on the basement membrane that extends its cytoplasm to the tubular lumen, forming extensive and intricate lateral and apical cytoplasmic processes that surround and attach to each germ cell through junctional proteins [23,64], providing structural and functional support to spermatogenesis [65]. Considering that the extensive surface area of the Sertoli cell (SC) plasma membrane maintains intimate contact with numerous germ cells, we propose that, following viral infection and replication, a single SC may transport viral particles to multiple germ cells at various stages of spermatogenesis. Consequently, it is plausible that SCs contribute to the dissemination of viral particles, a hypothesis supported by our observation of apparently normal germ cells surrounded by damaged SC processes. The presence of infected germ cells across different developmental phases—spanning from the basal to the adluminal compartments—further reinforces this mechanism. Furthermore, the capacity of SCs to phagocytose dying infected germ cells may exacerbate their own viral load, thereby promoting further transmission throughout the seminiferous epithelium.

### 3.2. SARS-CoV-2-Induced Pro-Inflammatory Response Impairs Junctional Proteins and Spermatogenesis

Our findings showed germ cell death and a decreased number of spermatocytes and Ki-67-immunopositive cells, confirming spermatogenic failure. The proliferation of spermatogonia [66] as well as meiotic progression [67] and late maturation [68,69,70,71] of spermatocytes depend on Cx43 integrity, which provides SCs-germ cells crosstalk. According to Weider et al. [71], intercellular communication through gap junctions is essential in regulating spermatogenesis. The loss of Cx43 inhibits spermatogenesis in adult mice [72]. Therefore, the low spermatogenic activity and germ cell death observed in IG may be caused, at least in part, by Cx43 downregulation. However, it is also important to emphasize that during COVID-19 progression, TNF-α and IFN-γ are the main cytokines involved in inflammatory cell death, impairing vital organs [41]. High levels of IFN-γ decrease the number of SCs and germ cells in adult mice [73] and induce cell cycle arrest and apoptosis in a model of ovarian cancer in vivo [74]. In orchitis, the overexpression of IFN-γ and TNF-α and the synergy between these cytokines impair spermatogenesis [75]. This same synergism has been associated with SARS-CoV-2-induced inflammatory responses [41] and has induced inflammatory cell death through the STAT1/IRF1 pathway [36,41,42]. According to Stephanou and Latchman [76], IFNγ-activated STAT1 induces apoptosis in a variety of cell types. During SARS-CoV-2 infection, TNF-α and IFN-γ synergy induces proliferative arrest and STAT1 hyperactivation, leading to hyperinflammation and activation of ACE2, which facilitates viral entry into host cells [61]. In the testes of K18-hACE2 animals, Giannakopoulos et al. [13] have also demonstrated that SARS-CoV-2 induces IFN-β, TNF-α, and IL-6 upregulation. Therefore, in the present study, the spermatogenic failure, including germ cell death, was likely induced by TNF-α and/or IFN-γ in the SARS-CoV-2-infected testes. The overexpression of *Stat1* in the infected testes, but not *Stat3* (related to cell survival and anti-inflammatory response), corroborates this finding.

The TNF-α and IFNγ-mediated STAT1/IRF1 pathway induces inflammatory cell death through the production of iNOS and NO [41], which depends on the activation of NF-κB, a crucial factor in the testicular inflammatory responses and cell death [77,78]. High levels of NO are necessary to combat viruses [41,79]. Cells infected by a virus, including epithelial cells, detect pathogen-associated molecular patterns (PAMPs), such as viral components/proteins, and trigger signaling pathways (STAT1) that activate transcription factors, such as NF-κB, which translocate to the nucleus and upregulate iNOS transcription [80]. Our findings showed strong nuclear NF-κB immunolabeling in the germ cells of IG, indicating that this factor was activated in these cells. This finding was corroborated by the enhanced epithelial iNOS immunolabeling and *iNOS* gene expression observed in IG. Oxidative stress impairs spermatogenesis by damaging the BTB and disrupting testicular cell functions [81], being one of the main causes of male infertility [82,83]. Another pathway involved in the production of iNOS is mediated by interferon-gamma (IFN-γ), which activates the Jak-1/STAT-1 axis and iNOS upregulation [84]. Therefore, the increased TNF-α, IFNγ, NF-κB, and iNOS immunoexpression observed in the SARS-CoV-2-infected seminiferous epithelium confirms a pro-inflammatory response of this epithelium to the viral infection.

After 5 days of infection, a high frequency of damaged seminiferous tubules showing intraepithelial spaces/vacuoles and detached germ cells was found. These changes were responsible for the significant reduction in total and epithelial areas observed in IG. In a study using “*post-mortem*” testes, SARS-CoV-2 was detected in SCs and spermatogonia associated with changes in the seminiferous epithelium [8]. Other types of viruses, such as HIV-1 [85,86], Zika virus (ZIKV) [16], and Hepatitis E virus [87], cause similar changes in the seminiferous tubules, impairing spermatogenesis.

SCs are susceptible to different viruses, such as Zika virus (ZIKV) [15,16], Marburg virus [14], mumps virus [18], and SARS-CoV-2 [8,9,19]. The infection by these viruses impairs the BTB and disrupts the structural and functional integrity of SCs, impairing spermatogenesis. ZIKV infection, for example, reduces the interaction between F-actin and ZO-1, enhancing BTB permeability [20], whereas mumps virus infection impairs BTB integrity through TLR2-mediated TNF-α production in SCs [18]. In SC’s culture, the expression of SARS-CoV-2 viral proteins, such as spike, impairs the expression of BTB proteins, such as ZO-1, N-cadherin, and Cx43 [21]. Our findings showed reduced Cx43 protein in the basal compartment of seminiferous epithelium as well as *Jam-α* downregulation, confirming that these BTB proteins were impaired following SARS-CoV-2 infection. These findings may be related to the significant increase in intratubular cytokines (TNF-α and INF-γ), observed in IG. High levels of TNF-α and INF-γ reduce Cx43 expression [88,89,90] and inhibit claudin-11 in SCs [30]. Moreover, in testicular autopsies of COVID-19 individuals, the intense expression of TNF-α was associated with reduced levels of BTB proteins, including Cx43 [16]. TNF-α high levels can also promote the endocytosis of the junctional protein Jam-α from the SCs membrane [31]. Moreover, *Jam-α* downregulation has been mediated by IFN-γ and TNF-α upregulation in renal cell carcinoma [91]. Since Jam proteins play essential roles in cell junction dynamics either in the BTB or in the Sertoli-germ cell interface [92], the impaired junctional proteins observed here may be caused by high cytokine levels, which explains the high incidence of germ cell death and reduction in epithelial area in IG. These epithelial changes corroborate a previous study, which demonstrated detached germ cells in the lumen of the proximal region of SARS-CoV-2-infected epididymis [56].

It is important to emphasize that, in a previous study using the same animals and treatment protocol, we demonstrated that SARS-CoV-2 infects Leydig cells and impairs steroidogenesis following 5 days of infection [10]. Thus, we cannot exclude the possibility that the low testosterone levels exacerbate the epithelial changes induced by the viral infection. In the current study, the inflammatory response seems to be the main cause of seminiferous epithelium damage as the epithelial changes typically take longer than 5 days to manifest following a drop in testosterone levels. Moreover, studies have demonstrated that the testicular changes and reduction in sperm concentration observed in infected mice are mitigated following the treatment with dexamethasone [93], reinforcing the idea that the inflammatory response is the main cause of spermatogenic failure.

## 4. Materials and Methods

### 4.1. Preparation of SARS-CoV-2 Samples

The B1 SARS-CoV-2 strain (SARS-CoV-2/human/BRA/SPBR-02/2020, GenBank Accession No. MT710714) used in this study was derived from COVID-19 patients at the Hospital of Ribeirão Preto, USP. This virus was subsequently propagated and titrated in Vero E6 cells within a BSL3 laboratory (Registration number: CBQ 0030/97) at the Ribeirão Preto Medical School. Cell cultures were maintained in DMEM enriched with 10% FBS and standard antibiotic/antimycotic agents (10,000 U/mL penicillin; 10,000 µg/mL streptomycin). For viral amplification, the inoculum was introduced to Vero cells cultured in DMEM supplemented with 2% FBS, followed by incubation for 48 h at 37 °C in a 5% CO_2_ atmosphere. The resulting cytopathogenic effects were monitored microscopically. Following observation, the cell monolayer was harvested, the supernatant collected and stored at −70 °C, and viral quantification performed using the plaque-forming unit (PFU) assay.

### 4.2. K18-hACE2 Transgenic Mice: Treatment and Viral Inoculation

To mimic human COVID-19, we utilized 12-week-old male K18-hACE2 mice (C57BL/6 background), a lineage known to replicate the clinical and histopathological features of the disease [94,95]. A total of 20 animals, originally from the Jackson Laboratory and bred at the FMRP/USP Animal Special Breeding Center, were equally divided into control (CG; n = 10) and infected (IG; n = 10) groups. Throughout the experiment, mice were housed under a 12 h light/dark cycle with controlled temperature (23 ± 2 °C), humidity (65–75%), and free access to food and water. Post-infection, the IG was maintained in a Biosafety Level 3 (BSL3) facility at FMRP/USP for 5 days. The animals were randomly housed (four per cage), regardless of their individual characteristics, before the experiment, and were properly identified to avoid confounders. The animals of IG were inoculated with 5 × 10^4^ PFU of SARS-CoV-2 (in 40 μL) by the intranasal route, whereas the control mice were inoculated with an equal volume of DMEM. This concentration was chosen based on the study by Dong et al. (2022) [46], which demonstrated that robust testicular infection in the K18-hACE2 model requires a high viral burden to overcome tissue-specific barriers. Moreover, our previous study [10] confirmed that this dose induces acute respiratory syndrome (COVID-19) and infects testicular cells. The weights and clinical signs were evaluated daily for 5 days after infection. The animals exhibiting different signals from those expected were excluded from the experiment. Since the animals begin to succumb at 7–8 days post-infection [95], the animals were euthanized 5 days post-infection to avoid animal suffering.

The care, use, and treatment of animals were followed according to ARRIVE guidelines 2.0, and the protocol of treatment used in this study was approved by the Ethical Committee for Animal Research of Dental School, UNESP, Araraquara, São Paulo, Brazil (protocol number 21/2022, 19 April 2022).

### 4.3. Histological Procedures

Following the 5-day experimental period, mice were weighed and anesthetized (80 mg/kg ketamine; 8 mg/kg xylazine; Virbac, Jurubatuba, Brazil). The right testes were fixed in 4% formaldehyde (buffered with 0.1 M sodium phosphate, pH 7.4) for 48 h before being processed for paraffin or historesin embedding. H&E staining was performed for morphological and morphometric analysis, whereas silanized slides were prepared for immunolabeling and detection of cell death (TUNEL). For molecular analysis (Western blot and qPCR), portions of the left testis were stored at −80 °C; additional fragments were fixed in Karnovsky’s solution for ultrastructural examination via electron microscopy.

### 4.4. Transmission Electron Microscopy (TEM) Processing

Testicular tissue (n = 3) was prepared for TEM according to previously described methods [96]. Samples were immersed in a 4% glutaraldehyde/4% formaldehyde fixative (0.1 M sodium cacodylate, pH 7.2) for 17 h at room temperature. Secondary fixation was performed in 1% osmium tetroxide for 1 h, followed by immersion in 2% aqueous uranyl acetate for 2 h. Dehydration was carried out in increasing ethanol concentrations, followed by propylene oxide clearance and Araldite embedding. Suitable regions for ultrastructural study were selected from 1% toluidine blue-stained semithin sections. Ultrathin sections were then placed on copper grids, contrasted with alcoholic uranyl acetate and lead citrate, and examined under an FEI TECNAI transmission electron microscope (Hillsboro, OR, USA).

### 4.5. Histopathological and Morphometric Analysis

The photomicrographs were obtained using a DP-71 camera (Olympus, Tokyo, Japan) attached to an Olympus BX-51 microscope (Tokyo, Japan). The morphometric analyses were performed using the Image Analysis System—Image Pro-Express 6.0 (Olympus, Tokyo, Japan). During all analyses, the samples were coded, and the researcher performing the analyses remained blinded to the group allocation until the statistical analyses were complete. The number of animals per group, for each morphometric analysis, was n = 6.

#### 4.5.1. Tubular Areas and Frequency of Abnormal Tubules

The seminiferous tubule (ST) size is variable according to the stage of the seminiferous cycle; thus, in an attempt to standardize the tubular sections to be measured, 15 ST exhibiting a round shape and at the specific stages of the seminiferous epithelium cycle—I–IV, V–VI, VII–VIII, and IX–XII [97] were randomly selected, totaling 60 tubules per animal. This approach ensures a robust and reliable standardization for accurate measurement, preventing bias. In each tubule, the area of the seminiferous epithelium and the area of the total tubular section (tubular area) were measured [98]. The luminal area was obtained by subtracting the epithelial area from the total area.

The number of ST showing abnormal epithelium, intraepithelial spaces, and/or containing sloughed germ cells in the lumen was quantified, and the frequency of these abnormal seminiferous tubules was calculated.

#### 4.5.2. Number of Sertoli Cells and Spermatocytes

As the number of germ cells and Sertoli cells is variable according to the stages of the seminiferous epithelium, the number of SCs and spermatocytes was quantified at specific stages in an attempt to standardize the quantification in both groups, avoiding bias.

Thus, in non-serial testicular sections from six animals per group, thirty-two ST per animal at stages VII–VIII and exhibiting a round shape were randomly selected, and the number of SC nuclei with typical morphology and evident nucleolus [99,100] was quantified. In the tubules at stages IX–XI exhibiting a round shape, the number of pachytene to diplotene spermatocytes was computed. These stages (IX–XI) were selected since they are post-spermiation stages whose epithelium is thinner than that of the other stages, and the pachytene to diplotene spermatocytes are easily identified. The number of SCs and spermatocytes per ST was calculated.

### 4.6. TUNEL Method

Apoptotic DNA fragmentation was detected using the TUNEL assay (Terminal deoxynucleotidyl transferase-mediated dUTP Nick-End Labeling) with the ApopTag^®^ peroxidase in situ kit (Millipore; Temecula, CA, USA) according to Beltrame et al. [100]. The endogenous peroxidase activity was inhibited with 3% hydrogen peroxide, followed by DNA end-labeling through incubation with the TdT enzyme. The fragments were then labeled using anti-digoxigenin-peroxidase antibodies, and the reaction was revealed with 0.06% 3,3-diaminobenzidine (DAB). Sections of mammary gland provided by the manufacturer of the kit were used as positive controls for the TUNEL method. Testicular sections, used as negative controls, were incubated in a TdT enzyme-free solution.

### 4.7. Immunohistochemistry and Immunofluorescence Reactions

Ki-67 (cell proliferation marker) and IFN-γ were detected by immunohistochemistry. hACE2 (human angiotensin-converting enzyme 2), spike and nucleocapsid proteins (viral proteins), TNF-α (pro-inflammatory cytokine), iNOS (inducible Nitric Oxide Synthase), connexin 43 (gap junction protein), and NF-kB (transcription factor) were detected by immunofluorescence.

Sections were immersed in 0.001 M citrate buffer (pH 6.0) and heated in a microwave oven at 95 °C for 30 min for antigen recovery. For the detection of Ki-67 and IFN-γ by immunohistochemistry, sections were previously immersed in hydrogen peroxide for endogenous peroxidase inactivation. All sections were incubated in 2% BSA for 30 min, and incubated at 4 °C overnight with the following primary antibodies: mouse anti-hACE2 monoclonal antibody (RRID: AB_2861379, 1:500, Santa Cruz Biotechnology, Dallas, TX, USA, SC-73668, lot: #G1222), rabbit anti-SARS-CoV-2 spike protein S1 recombinant monoclonal antibody (RRID: AB_2866477, 1:250, Invitrogen, Carlsbad, CA, USA, MA5-36247, lot: XG3635472), rabbit anti-SARS-CoV-2 nucleocapsid protein monoclonal antibody (1:3000; EPR24334-118; Abcam, Cambridge, UK; ab271180), rabbit anti-Ki-67 monoclonal IgG antibody (1:200; Abcam, Cambridge, UK; ab16667), and rabbit anti-IFN-γ polyclonal IgG antibody (1:300, Invitrogen, cat. 95560, lot: XH3666559); mouse anti-TNF-α monoclonal IgG [52B83] antibody (1:200, Abcam, Cambridge, UK; ab1793, lot:GR3446230), rabbit anti-iNOS recombinant polyclonal IgG [RM1017] antibody (1;1500; Abcam, Cambridge, UK; ab283655, lot:GR3436095-8), mouse anti-connexin 43 monoclonal antibody (RRID: AB_10707826, 1:200; Santa Cruz Biotechnology; sc-271837), and rabbit anti-NF-kB p65 polyclonal antibody ab31481 (1:200; Abcam, Cambridge, UK; ab31481). Sections incubated with anti-Ki-67 and anti-IFN-γ IgG antibodies were washed in PBS and incubated at room temperature with biotinylated anti-mouse and anti-rabbit IgG secondary antibody and peroxidase-labeled streptavidin (Universal Dako LSAB Kit, Dako Inc., Carpinteria, CA, USA, K4061, lot: 10136201). The reactions were stained with 3.3′-diaminobenzidine (DAB: Dako Liquid DAB + Substrate Chromogen system, Dako Inc., Carpinteria, CA, USA, K3468, lot: 10147082), counterstained with Carazzi’s haematoxylin, and mounted with Permount^®^ resin mounting medium.

The testicular sections subjected to immunofluorescence reactions were washed in PBS and incubated in the dark with the following secondary antibodies: Alexa Fluor^®^488 anti-mouse IgG antibody (1:1000; Molecular Probes^®^ by Life Technologies, Carlsbad, CA, USA, A11001, lot: 1664729) and Alexa Fluor^®^594 anti-rabbit IgG antibody (1:500; Invitrogen^®^ by Thermo Fisher Scientific, Carlsbad, CA, USA, R3117, lot:2086924), for 1 h at room temperature. After washing in PBS, nuclear staining was performed with DAPI (1:500, Molecular Probes by Life Technologies; Carlsbad, CA, USA, R37606, lot:1616913) for 5 min in the dark at room temperature. The slides were mounted with Fluoromount^®^ mounting medium (Dako faramount Aqueous mounting medium, Dako Inc., Carpinteria, CA, USA, S3025, lot: 11176284) or Fluro-Gell III Mounted Medium^®^ (Electron Microscopy Sciences, Cat.#17985-60, Lot.#180618, Hatfield, PA, USA). To check possible nonspecific binding of the secondary antibodies to the tissues, negative controls were performed by incubating sections with non-immune serum instead of primary antibodies.

### 4.8. Number of Ki-67 Positive Cells

In non-serial testicular sections (distance between sections around 30 µm) of six animals per group, thirty round-shaped ST at stages IX–XI, which contain a typical layer of pachytene to diplotene spermatocytes (as described above—item 4.5.2), were randomly selected under the Olympus BX-51 microscope (Tokyo, Japan) equipped with a DP-71 camera (Olympus, Tokyo, Japan). In each tubular section, the number of Ki-67-immunopositive germ cells (spermatogonia and spermatocytes) was quantified. The number of Ki-67-immunopositive cells per ST was calculated.

### 4.9. Double Immunofluorescence Analysis

To confirm the presence of hACE2 and the SARS-CoV-2 infection in the testicular cells, double immunofluorescence was performed to detect hACE2 + spike as well as vimentin (Sertoli cell marker) + nucleocapsid. The double immunofluorescence reactions were performed according to de Santi et al. [101]. After antigen recovery, the sections were incubated overnight at 4 °C with mouse anti-human ACE2 monoclonal antibody (RRID: AB_2861379, 1:500, Santa Cruz Biotechnology, Dallas, TX, USA, SC-73668, lot: #G1222) or mouse anti-vimentin monoclonal antibody (RRID: AB_261856, 1:30; V9; Sigma-Aldrich, St. Louis, MO, USA; V2258). The day after, the sections were washed and incubated with Alexa Fluor^®^488 anti-mouse antibody (1:1000; Molecular Probes^®^ by Life Technologies, Carlsbad, CA, USA, A11001, lot: 1664729) for 1 h at room temperature. After washing in PBS, the sections were incubated overnight at 4 °C, with rabbit anti-SARS-CoV-2 spike protein S1 recombinant monoclonal antibody (RRID: AB_2866477, 1:250, Invitrogen, MA5-36247, lot: XG3635472) or rabbit anti-SARS-CoV-2 nucleocapsid protein monoclonal antibody (1:3000; EPR24334-118; Abcam, Cambridge, MA, USA; 215 ab271180). The day after (third day), the sections were washed in high-salt PBS and incubated in Alexa Fluor^®^594 anti-rabbit IgG antibody (1:500; Invitrogen^®^ by Thermo Fisher Scientific, Carlsbad, CA, USA,: R3117, lot:2086924) for 1 h at room temperature. After washing in PBS, nuclear staining was performed with DAPI (1:500, Molecular Probes by Life Technologies; Carlsbad, CA, USA, R37606, lot:1616913) for 5 min in the dark at room temperature, and the slides were mounted with Fluoromount^®^ mounting medium (Dako Faramount Aqueous mounting medium, Dako Inc., Carpinteria, CA, USA, S3025, lot: 11176284). Negative controls were performed following the same protocol and steps, except that the primary antibodies were replaced by non-immune serum.

### 4.10. Immunofluorescent Areas

Immunofluorescent areas were analyzed using a DFC 550 Camera (Leica, Wetzlar, Germany), connected to a BM4000 B LED microscope (Leica, Wetzlar, Germany), and the Leica Application Suite software (LAS 4.3, Leica, Wetzlar, Germany). All software parameters were rigorously standardized, ensuring that only areas displaying intense fluorescence were considered.

In non-serial testicular sections of six animals per group, the immunofluorescent areas, corresponding to each marker analyzed, were measured in a standardized total epithelial area. The immunofluorescent areas/mm^2^ of seminiferous epithelium were calculated.

To guarantee standardization of the seminiferous tubules, which are at different stages of the seminiferous epithelium cycle, the immunofluorescent areas of TNF-α, IFN-γ, and Cx-43 were measured in 15 tubular sections at stages IX–XI. Each tubular area was measured, and the immunofluorescent areas/mm^2^ of seminiferous epithelium were calculated [98].

### 4.11. Western Blot

Testicular tissue samples (n = 4 per group) were homogenized in a lysis buffer (50 mM Tris pH 8.0, 150 mM NaCl, 1 mM EDTA, 10% glycerol, 1% Triton X-100) supplemented with 1 mM PMSF and a protease inhibitor cocktail (5 ng/mL each of Pepstatin, Leupeptin, Aprotinin, Antipain, and Chymostatin; Sigma-Aldrich; St. Louis, MO, USA). After an overnight incubation at 4 °C and subsequent centrifugation (8944 g for 20 min), protein levels in the supernatants were determined using the Bradford method (Sigma-Aldrich). Protein aliquots (40 µg) were resolved via 10% SDS-PAGE and transferred onto 0.2 µm nitrocellulose membranes (Bio-Rad; Hercules, CA, USA). Nonspecific binding was blocked for 1 h with 5% nonfat dry milk in TBS/T, followed by overnight incubation at 4 °C with primary antibodies against TNF-α (1:200; Abcam, Cambrige, UK; RRID: AB_302615) and angiotensin II (Abcam). After TBS/T washes, membranes were incubated with an HRP-conjugated secondary antibody (1:9000; Sigma-Aldrich) for 1 h at room temperature. Immunoreactive bands were detected using an enhanced chemiluminescence system (ECL; Boster; Pleasanton, CA, USA). For normalization, membranes were stripped and re-probed with an anti-β-tubulin antibody (1:8000; Sigma-Aldrich). Total protein loading was verified by Ponceau staining, and optical density (OD) quantification was performed using Image Lab software version 3.0 (Bio-Rad; Hercules, CA, USA). Statistical analysis and normalization were conducted using GraphPad Prism 6.0, with all samples processed in triplicate.

### 4.12. Reverse Transcription and Real-Time Polymerase Chain Reaction (RT-qPCR)

The primer design was performed using the murine sequences available at the University of California, Santa Cruz (UCSC) Genome Browser and the Primer3 program [102] (Table 2). Testis fragments from five animals per group were immersed in RNA Keeper stabilizing reagent (LGC Biotecnologia, Cotia, Brazil; 14-0002-01) and stored at −80 °C. Testis sample RNA was isolated and purified using the Aurum Total RNA Mini Kit (Bio-Rad Laboratories, Hercules, CA, USA; 732-6820). The cDNA was obtained using the High-Capacity cDNA Reverse Transcription Kit (Applied Biosystems, Cheshire, UK; 4368814) according to the manufacturer’s protocol. The real-time PCR was performed using the QuantStudio 3 Real-Time PCR instrument (Applied Biosystems, ThermoFisher; Life Technologies Holdings, Waltham, MA, USA) and the PowerUp SYBR Green Master Mix (Applied Biosystems, Cheshire, UK; A25742). The qPCR cycling conditions were as follows: 40 cycles of denaturation at 95 °C for 15 s, annealing and extension at 60 °C for 1 min, and a final extension step with a ramp rate of 0.15 °C/s at 95 °C for 15 s. For gene expression analysis, the results were reported as mean  ±  SD, using the formula ΔCt  =  [Ct target gene − Ct housekeeping gene β-actin]. Relative expression is derived from log(2^−ΔΔCt^), where ΔΔCt  =  ΔCt testes of IG—mean of ΔCt control group.

### 4.13. Statistical Analysis

Morphometric data were analyzed using GraphPad Prism^®^ 8.4.3 software (GraphPad Software, CA, USA). The Kolmogorov–Smirnov test was applied to assess data normality. Based on the distribution profile of the data, differences between the control and infected groups were evaluated using the unpaired Student’s *t*-test, assuming significance at *p* ≤ 0.05. The results were expressed as means ± SD in box and whisker plots.

## 5. Conclusions

The seminiferous epithelium of K18-hACE2 transgenic mice was infected with SARS-CoV-2, and high hACE2 and angiotensin II levels may contribute to the infection, enhancing virulence in the seminiferous epithelium. In this tissue, the viral infection triggered a localized release of TNF-alpha and IFN-gamma, culminating in NF-kB- (and possible STAT1)-induced iNOS overexpression. The resulting oxidative stress, combined with the impaired BTB, was responsible for the germ cell death and low mitotic/meiotic activity, impairing spermatogenesis.

The findings also indicate that the presence of SARS-CoV-2 spike and nucleocapsid proteins in Sertoli cells, associated with assembled viral particles in these cells, provides evidence of viral invasion of these cells, which initiate TNF-alpha-induced immune response, culminating in BTB disruption and pro-apoptotic signaling. Moreover, the intricate cytoplasmic projections of these sustentacular (nurse) cells, combined with their capacity to engulf dying infected germ cells, may favor the dissemination of viruses throughout the epithelium.

## Figures and Tables

**Table 1:** Body weight (BW), absolute testicular weight (ATW), total tubular area (TTA), seminiferous epithelium area (SEA), and tubular luminal area (TLA) in CG and IG.

	BW (g)	ATW (g)	TTA (µm^2^)	SEA (µm^2^)	TLA (µm^2^)
**CG**	26.20 ± 0.59	0.1050 ± 0.0022	37,774 ± 532.6	36,476 ± 527.1	1298 ± 106.8
**IG**	23.20 ± 1.02 *	0.1467 ± 0.0255	32,953 ± 1415 *	26,871 ± 2241 *	6082 ± 1298 *

* *p* < 0.05.

**Table 2:** Sequence of primers used in qPCR.

Gene	References	Length (bp)	Oligonucleotide Sequences (5′-3′)	Tm
*Stat1* (Exxtend, Brazil)	[103]	21 20	F: CACCCTTGCTTACTCTACTGC R: TTGAATGACTAAACGCCTGA	60.0° 60.0°
*Stat3* (Exxtend, Brazil)	[104]	20 19	F: TATGGTCCTTATTCTATGCG R: CAGACAGTTGCCAGTCTCA	56.0° 58.0°
*Jam-α* (Exxtend, Brazil)	[105]	21 20	F: GGTCAGCATCCACCTCACTGT R: AGGTCAGCACTGCCCTGTTC	60.0° 60.0°
*iNOS* (Exxtend, Brazil)	[106]	22 21	F: CTCACCTACTTCCTGGACATTAC R: CAATCTCTGCCTATCCGTCTC	60.0° 60.0°
*β-Actin* (Exxtend, Brazil)	[56]	18 20	F: CTGCGCTTCCTTTGTCCC R: GACAATTGAGAAAGGGCGTG	57.0° 55.0°

## Data Availability

No new data were created or analyzed in this study. Data sharing is not applicable to this article.
